# Anorexia nervosa and cancer: a protocol for a systematic review and meta-analysis of observational studies

**DOI:** 10.1186/s13643-017-0540-5

**Published:** 2017-07-11

**Authors:** Ferrán Catalá-López, Brian Hutton, Jane A. Driver, Manuel Ridao, José M. Valderas, Ricard Gènova-Maleras, Jaume Forés-Martos, Adolfo Alonso-Arroyo, Diego Macías Saint-Gerons, Eduard Vieta, Alfonso Valencia, Rafael Tabarés-Seisdedos

**Affiliations:** 1 Department of Medicine, University of Valencia/INCLIVA Health Research Institute and CIBERSAM, Valencia, Spain; 2 Fundación Instituto de Investigación en Servicios de Salud, Valencia, Spain; 3 Clinical Epidemiology Program, Ottawa Hospital Research Institute, Ottawa, Ontario Canada; 4 School of Epidemiology, Public Health and Preventive Medicine, University of Ottawa, Ottawa, Ontario Canada; 5 Geriatric Research Education and Clinical Center, VA Boston Healthcare System, Boston, MA USA; 6 Division of Aging, Department of Medicine, Brigham and Women’s Hospital, Harvard Medical School, Boston, MA USA; 7 Instituto Aragonés de Ciencias de la Salud, Red de Investigación en Servicios de Salud en Enfermedades Crónicas (REDISSEC), Zaragoza, Spain; 8 Fundación para el Fomento de la Investigación Sanitaria y Biomédica de la Comunitat Valenciana (FISABIO-Salud Pública), Valencia, Spain; 9 Health Services and Policy Research Group, Exeter Collaboration for Academic Primary Care, University of Exeter Medical School, University of Exeter, Exeter, UK; 10 Directorate General for Public Health, Regional Health Council, Madrid, Spain; 11 Department of History of Science and Documentation, University of Valencia, Valencia, Spain; 12 Unidad de Información e Investigación Social y Sanitaria-UISYS, University of Valencia-Spanish National Research Council (CSIC), Valencia, Spain; 13 Division of Pharmacoepidemiology and Pharmacovigilance, Spanish Medicines and Healthcare Products Agency, Madrid, Spain; 14 Department of Health Systems and Services, Unit of Medicines and Health Technologies, Pan American Health Organization, Washington, DC, USA; 15 Hospital Clínic, Universitat de Barcelona, Institut d’Investigacions Biomèdiques August Pi i Sunyer (IDIBAPS) and CIBERSAM, Barcelona, Spain; 16 Life Sciences Department, Barcelona Supercomputing Center, Barcelona, Spain; 17 Structural Biology and Biocomputing Programme, Spanish National Cancer Research Centre (CNIO), Madrid, Spain

**Keywords:** Anorexia nervosa, Cancer, Eating disorder, Epidemiological study, Systematic review, Meta-analysis

## Abstract

**Background:**

Anorexia nervosa is characterized by a severe restriction of caloric intake, low body weight, fear of gaining weight or of becoming fat, and disturbance of body image. Pathogenesis of the disorder may include genetic predisposition, hormonal changes and a combination of environmental, psychosocial, and cultural factors. Cancer is the second leading cause of death worldwide. At present, no systematic reviews and meta-analyses have evaluated the risk of cancer in people with anorexia nervosa. The objective of this study will be to evaluate the association between anorexia nervosa and the risk of developing or dying from cancer.

**Methods/design:**

This study protocol is part of a systematic collection and assessment of multiple systematic reviews and meta-analyses (umbrella review) evaluating the association of cancer and multiple central nervous system disorders. We designed a specific protocol for a new systematic review and meta-analysis of observational studies of anorexia nervosa with risk of developing or dying from any cancer. Data sources will be PubMed, Embase, Scopus, Web of Science, and manual screening of references. Observational studies (case–control and cohort) in humans that examined the association between anorexia nervosa and risk of developing or dying from cancer will be sought. The primary outcomes will be cancer incidence and cancer mortality in association with anorexia nervosa. Secondary outcomes will be site-specific cancer incidence and mortality, respectively. Screening of abstracts and full texts, and data abstraction will be performed by two team members independently. Conflicts at all levels of screening and abstraction will be resolved through discussion. The quality of studies will be assessed by using the Ottawa-Newcastle scale by two team members independently. Random effects models will be conducted where appropriate. Subgroup and additional analyses will be conducted to explore the potential sources of heterogeneity. The World Cancer Research Fund (WCRF)/American Institute for Cancer Research (AICR) criteria and the Grading of Recommendations Assessment, Development and Evaluation (GRADE) approach will be used for determining the quality of evidence for cancer outcomes.

**Discussion:**

Findings from this systematic review will inform an ongoing umbrella review on cancer and central nervous system disorders. Our systematic review and meta-analysis of observational studies will establish the extent of the epidemiological evidence underlying the association between anorexia nervosa and cancer.

**Systematic review registration:**

PROSPERO CRD42017067462.

**Electronic supplementary material:**

The online version of this article (doi:10.1186/s13643-017-0540-5) contains supplementary material, which is available to authorized users.

## Background

Anorexia nervosa is a mental disorder characterized by a severe restriction of caloric intake, a significantly low body weight for the developmental stage, an intense fear of gaining weight or of becoming fat, and a severe disturbance of body image. Pathogenesis of the disorder may include genetic predisposition, hormonal changes, and a combination of environmental, psychosocial, and cultural factors [1–3]. Anorexia nervosa can affect people of all ages and genders, and have been reported worldwide both in high income and low-middle income regions [4–6]. Most recent burden of disease estimates revealed 2.9 million people with anorexia nervosa (representing 653,019 disability-adjusted life years lost) around the world [5, 6]. The disorder is more prevalent among adolescent and young women; however, young men may also be affected [5]. Although most patients eventually recover, anorexia nervosa can blight young lives and distort development [7–10].

Cancer is the second leading cause of death worldwide [11, 12], with over 8.8 million deaths in 2015 [12]. There is evidence suggesting that excess body weight is a risk factor for several cancers. For example, a recent umbrella review of 204 meta-analyses [13] found strong evidence for the association between body mass index and cancers of digestive organs (esophageal adenocarcinoma and cancers of the colorectum, biliary tract system, and pancreas), hormone-related cancers (such as breast cancer in women), endometrial cancer, kidney cancer, and multiple myeloma; but also between adiposity and the risk of colorectal cancer, gallbladder cancer, gastric cardia cancer, ovarian cancer, and multiple myeloma [13]. The underlying mechanisms between excess body weight and cancer are complex and are not yet fully understood. Excess body weight and adiposity might affect immune system function and inflammatory processes, levels of certain hormones (such as insulin and estrogen), factors that regulate cell growth (such as insulin-like growth factor-1 [IGF-1]), and proteins that influence how the body uses certain hormones (such as sex hormone-binding globulin), among others [14–16].

Research on how losing body weight might lower the risk of developing cancer is limited. Energy restriction (or calorie restriction) has been found to be protective against the development of cancer in experimental animal studies [17–20]. Energy restriction is difficult to study in human populations. Anorexia nervosa, an excessive form of calorie restriction associated with pathological weight loss, has been proposed as a biomarker of energy restriction [21, 22]. Several epidemiological studies [22–25] have evaluated whether there exists a general reduction in cancer development among patients with anorexia nervosa (the so-called, “energy-restriction hypothesis” [26]). For example, a retrospective cohort study by Mellemkjaer et al. [22] suggested a potential reduction, but not statistically significant, in cancer incidence among women with anorexia nervosa compared with the general population (standardized incidence ratio [SIR] = 0.80; 95% confidence interval [CI] 0.52–1.18). In another retrospective cohort study, Michels and Ekbom [23] reported that women hospitalized for anorexia nervosa were associated with a lower incidence of breast cancer compared to the general population (SIR = 0.47; 95% CI 0.19–0.97), with a larger effect among parous women (SIR = 0.24; 95% CI 0.03–0.87). However, a recent observational study assessing the risk of cancer among people with anorexia nervosa has suggested that the potential associations in humans remain controversial [27].

At present, no systematic reviews and meta-analyses have evaluated the epidemiological evidence examining the association between anorexia nervosa and cancer. To better understand the body of the evidence, we will conduct a systematic review and meta-analysis of observational studies in order to synthesize and evaluate the validity of the association between anorexia nervosa and the risk of developing or dying from cancer.

## Methods

### Protocol

This study protocol is part of an ambitious ongoing umbrella review (a systematic collection and assessment of multiple systematic reviews and meta-analyses) into the association of cancer and multiple central nervous system disorders [28]. To our knowledge, no previous systematic reviews and meta-analyses have evaluated the risk of developing or dying from cancer in anorexia nervosa. For this reason, we have designed a specific protocol for a new systematic review and meta-analysis. The present protocol has been registered within the PROSPERO database (registration number: CRD42017067462) and is being reported in accordance with the reporting guidance provided in the Meta-analysis Of Observational Studies in Epidemiology (MOOSE) reporting guideline [29] and the Preferred Reporting Items for Systematic Reviews and Meta-Analyses Protocols (PRISMA-P) statement [30, 31] (see PRISMA-P checklist in Additional file 1).

### Ethics

No ethical approval is required for the performance of this work.

### Search methods

We will systematically search, from inception, PubMed/MEDLINE, EMBASE, SCOPUS, and Web of Science to identify observational studies examining association between cancer and anorexia nervosa. A date restriction will not be imposed. The final electronic search strategies will be defined by a senior information specialist (AA-A) and by a clinical epidemiologist (FC-L). Keywords related to anorexia nervosa, cancer, and epidemiological studies will be used. A draft search strategy for PubMed/MEDLINE database has been included in Additional file 2. The reference lists of examined full-text papers will be scrutinized for additional relevant publications. We will also contact authors of primary publications and/or collaborators to check if they are aware of any studies we may have missed. There will be no restriction by language of publication, and we will arrange for translation where necessary.

### Eligibility criteria

Studies will be selected according to the following criteria: study design, participants (exposure), comparator(s) or control group, and outcome(s) of interest.

Study design: eligible studies will be observational studies reporting study specific data for cancer outcomes in people with anorexia nervosa. Prospective cohort studies, retrospective cohort studies (also known as historical cohort studies), and case–control studies will be included. Randomized controlled trials will be unavailable for our research question. We will exclude studies in which anorexia nervosa is not the exposure of interest, and cancer is not reported as the outcome of interest. Observational studies not presenting study specific data (e.g., relative risks, 95% confidence intervals, numbers of cases/population, observed and expected cases) or sufficient data for an outcome measure to be calculated will be also excluded. There will be no restriction by study setting.

Participants (exposure): index subjects will be patients with anorexia nervosa (regardless of age or sex). We will use investigator-reported definitions (according to accepted diagnostic criteria such as the International Classification of Diseases [ICD] or the Diagnostic and Statistical Manual of Mental Disorders [DSM] criteria: ICD-9: 307.1, 307.54; ICD-10: F50.0-F50.1). Exclusion criteria: animals, in vitro, and in vivo experiments.

Comparator(s) or control group: the comparator group will be based on subjects with no history of anorexia nervosa (e.g., the general population, the community, unexposed outpatient, or hospital-based controls).

Outcome(s): the primary outcomes will be cancer incidence and cancer mortality (all malignant neoplasms; ICD-9: 140–209; ICD-10: C00-C97) in association with anorexia nervosa. Given the varied biology of cancers, the risk of incident site-specific cancers, and the risk of fatal site-specific cancers will be explored as secondary outcomes. Site-specific cancers will be defined in groups that include ICD codes pertaining to neoplasms [11] (see Additional file 3).

### Screening and selection procedure

Two reviewers will screen all articles identified from the search independently. First, titles and abstracts of articles returned from initial searches will be screened based on the eligibility criteria outlined above. Second, full texts will be examined in detail and screened for eligibility. Third, references of all considered articles will be hand-searched to identify any relevant report missed in the search strategy by two reviewers independently. Any disagreement between reviewers will be resolved by discussion to meet a consensus.

### Data collection process

From each eligible observational study, two reviewers will independently extract information on first author, year of publication, epidemiological design (cohort or case–control, prospective, or retrospective), country of study, follow-up period, setting (mixed, inpatient, outpatient, or community), coverage (multi-center or single center study), general characteristics of participants (age, sex, ethnicity, and parity status), sample size, the outcomes of interest (including definitions and confounding factors that were taken into consideration), the number of cases and controls (in case–control studies) or the number of cases and population participants (in cohort studies) and/or the maximally adjusted relative risk (reported as odds ratio for case–control studies and hazard ratio or standardized incidence/mortality ratio for cohort studies), and 95% confidence intervals. We will use pre-designed forms that will be piloted initially on a small number of included reviews and observational studies. We will also contact authors of primary publications and/or collaborators for missing outcome data or unclear information.

### Quality and risk of bias assessment

The methodological quality and bias of primary epidemiological studies will be evaluated using the Newcastle-Ottawa scale (NOS) for observational studies [32]. Using the NOS tool, each study is judged on eight items, categorized into three groups: the selection of the study groups, the comparability of the groups, and the ascertainment of either the exposure or outcome of interest for case–control or cohort studies, respectively. Stars are awarded for each item, and the highest quality (low risk of bias) studies are awarded up to nine stars. We will consider studies with 0–3, 4–6, and 7–9 stars to represent low, moderate, and high quality, respectively. The quality (risk of bias) for each observational study will be independently assessed by two reviewers. Discrepant scores will be resolved by discussion and consensus.

### Methods for evidence synthesis

The data from each paper (e.g., population, study characteristics, outcomes, and findings) will be used to build evidence tables. Data from primary observational studies will be used to perform random-effects meta-analyses. We will estimate the summary effect size and its 95% confidence interval using the inverse variance method based on the DerSimonian and Laird random effects model [33]. The random-effects model is selected a priori to synthesize the epidemiological data, as it considers both within-study and between-study variation by incorporating the heterogeneity of effects into the overall analyses. We will evaluate heterogeneity by estimating the variance between studies using Cochran’s Q test [34] and *I* ^2^ statistic [35]. The Cochran’s Q test is obtained by the weighted sum of the squared differences of the observed effect in each study minus the fixed summary effect. The *I* ^*2*^ statistic is the ratio of variance between studies over the sum of the variances within and between studies, and ranges between 0 and 100% (with values of 0–25% and 75–100% taken to indicate low and considerable heterogeneity, respectively). In addition, we will calculate the 95% prediction interval [36, 37], which further accounts for between-study heterogeneity and evaluates the uncertainty for the effect that would be expected in a new observational study.

We will apply a set of criteria to conclude whether the evidence for a cancer outcome may be considered convincing, probable, limited-suggestive, limited-not conclusive, or unlikely. As described elsewhere [28], we will follow the Global Burden of Disease Study approach based on World Cancer Research Fund (WCRF)/American Institute for Cancer Research (AICR) criteria for grading the quality of evidence [38–40]. “Convincing evidence” consists of biologically plausible associations between exposure and outcome based on multiple epidemiological studies in different populations. Evidentiary studies must be substantial, include prospective observational studies, and where relevant, epidemiological studies of sufficient size, duration, and quality and showing consistent effects. A convincing relationship should be robust enough to be highly unlikely to be modified in the foreseeable future as new evidence accumulates. “Probable evidence” is similarly based on epidemiological studies with consistent associations between exposure and outcome but with existing shortcomings, such as insufficient prospective observational studies available. “Limited-suggestive evidence” represents too limited evidence to conclude on a probable or convincing causal association, but where there is evidence suggestive of a direction of effect. “Limited-not conclusive evidence” consists of information that is so limited that no firm conclusion can be made for several reasons (e.g., the evidence might be limited by the amount of evidence in terms of the number of studies available, by inconsistency of direction of effect, by poor quality of studies, or by any combination of these factors). “Substantial effect on risk unlikely” consists of evidence strong enough to support a judgment that a particular exposure is unlikely to have a substantial causal relation to a cancer outcome. The evidence should be robust enough to be unlikely to be modified in the foreseeable future as new evidence accumulates [40]. We will also use the Grading of Recommendations Assessment, Development, and Evaluation (GRADE) methodology for evaluating the quality of evidence for each outcome [41–43]. For purposes of systematic reviews, the GRADE approach defines the quality of a body of evidence as the extent to which one can be confident that an estimate of effect or association is close to the quantity of specific interest. Using GRADE, the quality of a body of evidence involves consideration of within-study risk of bias (methodological quality), directness of evidence, heterogeneity, precision of effect estimates, and risk of small study effects (sometimes called “publication bias”) [41–43]. GRADE rating will be adjudicated as high (further research is very unlikely to change our confidence in the estimate of effect), moderate (further research is likely to have an important impact on our confidence in the estimate of effect and may change the estimate), low (further research is very likely to have an important impact on our confidence in the estimate of effect and is likely to change the estimate), or very low (very uncertain about the estimate of effect) [41, 44].

### Additional analyses

If sufficient studies are identified, potential sources of heterogeneity will be investigated further by subgroup or meta-regression analyses according to baseline characteristics and methodological factors [28]. We plan to conduct subgroup analyses by sex (men or women), age (e.g., at first diagnosis of anorexia nervosa), study design (cohort or case–control; prospective, or retrospective), follow-up (0–1, >1–5, or >5 years), setting (mixed, inpatient, outpatient, or community), ethnicity (e.g., Asian or non-Asian), population-based (yes or no), country economic status (developed or developing countries according to International Monetary Fund), year of publication (before 2000 or in 2000 and after), study quality (high or low-moderate risk of bias), adjustment for confounding variables (age, sex or other), and sample size (<500, 500–1000 or >1000 participants). If sufficient information is identified, we will conduct subgroup analyses or meta-regression analyses for cancer types according to relationship with smoking (smoking-related cancer sites or other cancer sites) or sex hormones (cancers occurring in hormone-sensitive tissues such as breast, ovary, uterine endometrium, prostate and colorectal, where sex hormones exert an important role in cancer etiopathogenesis and progression) [23, 45, 46] (see Additional file 2). We will conduct a specific subgroup analysis among women based on parity status (parous or nulliparous women) [23, 24]. If sufficient studies are identified, we will perform cumulative meta-analyses in the order of publication year showing the consistency of evidence over time [47, 48]. Small study effects will be assessed by inspection of the funnel plots for asymmetry and with Egger’s test [49] and Begg’s test [50], with the results considered to indicate potential small study effects when *P* < 0.10.

### Software considerations

All analyses will be conducted in Stata version 13 or higher (StataCorp LP, College Station, Texas, USA) using the *metan* (for fixed and random effects meta-analysis), *metareg* (for meta-regression analysis), *metacum* (for cumulative meta-analysis), and *metabias* and *metafunnel* (for small study effects analysis) [51].

## Discussion

The systematic review presented in this protocol will inform an ongoing umbrella review and meta-analysis of observational studies on cancer and central nervous system disorders [28]. This systematic review will establish the extent of the epidemiological evidence underlying the association between anorexia nervosa and the risk of developing or dying from cancer, in a reproducible and rigorous way. The systematic review and meta-analysis presented in this protocol will be reported in accordance with the reporting guidance provided in the PRISMA statement [52] and the MOOSE reporting guideline [29]. Any amendments or modifications made in the protocol will be outlined and reported in the final paper.

Direct and inverse cancer comorbidity could be a relevant model to investigate common or related pathways or processes and test new therapies and prevention programs, but, most importantly, to understand why certain people might potentially be protected from the malignancy [25, 53, 54]. In this context, understanding the complex connections between anorexia nervosa and cancer might be important for clinical research and practice.

There are several strengths and limitations of our planned methods. We will comprehensively evaluate epidemiological data characterizing the associations between anorexia nervosa and cancer, exploring the extent of heterogeneity and bias in observational studies. We have planned assessments of meta-bias and strength of evidence statements. We anticipate that we will identify knowledge gaps to be filled by new research considering that some outcomes will be poorly covered in the biomedical literature. A key challenge is that based on knowledge from previous reviews on cancer and central nervous system disorders [54–57], we anticipate identifying studies using different study designs, populations, durations, and with a variable quality of reporting methods and results.

## Additional files

Additional file 1: PRISMA-P Checklist. (DOCX 27 kb)

Additional file 2: Key terms for PubMed/MEDLINE search. (DOCX 22 kb)

Additional file 3: Definitions of specific cancer-site outcomes. (DOCX 27 kb)

## Abbreviations

AICR: American Institute for Cancer Research
AMSTAR: Assessment of Multiple Systematic Reviews
DSM: Diagnostic and Statistical Manual of Mental Disorders
GRADE: Grading of Recommendations Assessment, Development, and Evaluation
ICD: International Classification of Diseases
MOOSE: Meta-analysis Of Observational Studies in Epidemiology
NOS: Newcastle-Ottawa scale
PRISMA-P: Preferred Reporting Items for Systematic Reviews and Meta-Analyses extension for Protocols
WCRF: World Cancer Research Fund
