# Supplementary material for: Anorexia nervosa and cancer: a protocol for a systematic review and meta-analysis of observational studies
Source: Syst Rev. 2017 Jul 11;6:137. doi: 10.1186/s13643-017-0540-5 (PMC5504548; doi:10.1186/s13643-017-0540-5)
Supplement: Supplementary file 2 — Key terms for PubMed/MEDLINE search. (DOCX 22 kb) [file 13643_2017_540_MOESM2_ESM.docx]

**Additional file 2**

**Table.** Key terms for PubMed/MEDLINE search.

| Search | Query |
| --- | --- |
| #1 | “(anorexia nervosa OR eating disorder*)” |
| #2 | “(cancer* OR carcinoma* OR neoplasia* OR tumor* OR neoplasm* OR maligna*)” |
| #3 | “(epidemiology OR epidemiologic* OR cohort stud* OR longitudinal stud* OR case-control stud*)” |
| #4 | #1 AND #2 AND #3  No limits |
